# Dr. Wm. Everett Palmer

**Published:** 1917-01

**Authors:** 


**Dr. Wm. Everett Palmer,** P. & S. 1882, died at his home in Hornell, Dec. 11, of apoplexy., aged 78.

